# Effect of method of administration on longitudinal assessment of quality of life in gynecologic cancer: An exploratory study

**DOI:** 10.1186/1477-7525-3-6

**Published:** 2005-01-17

**Authors:** Karen M Gil, Heidi E Frasure, Michael P Hopkins, Eric L Jenison, Vivian E von Gruenigen

**Affiliations:** 1 Department of Obstetrics and Gynecology, Akron General Medical Center, Akron, Ohio, USA; 2 Northeastern Ohio Universities College of Medicine, Rootstown, Ohio, USA; 3 Department of Obstetrics and Gynecology, University Hospitals of Cleveland, Cleveland, Ohio, USA

## Abstract

**Background:**

Longitudinal assessments of quality of life are needed to measure changes over the course of a disease and treatment. Computer versions of quality of life instruments have increased the feasibility of obtaining longitudinal measurements. However, there remain occasions when patients are not able to complete these questionnaires. This study examined whether changes measured using a computer version of the Functional Assessment of Cancer Therapy – General (FACT-G) on two occasions would be obtained if patients completed a paper version on one of the two occasions.

**Methods:**

Gynecologic oncology patients completed a computer version of the FACT-G pre-operatively and at six months. Patients were given the option of using the paper version instead of the computer at either time point. Repeated measures analysis of variance was used.

**Results:**

One hundred nineteen patients completed the FACT-G at both time points. Seventy-one (60%) patients used the computer at both visits, 26 (21.8%) used the computer followed by the paper version, 17 (14.3%) used the paper version followed by the computer version, and five patients (4.2%) used the paper version at both visits. Significant effects over time were obtained in the physical, functional, and emotional well-being domains, and in total scores, but there were no effects of method of administration of the questionnaires and no interaction between method of administration and changes over time.

**Conclusions:**

These data indicate that women are responding to the content of the questionnaire and not method of data collection. Although using the same method of administration of instruments over time is desirable, using alternate methods is preferable to forgoing data collection entirely. Large scale studies should be conducted to determine if the multiple methods of data collection that are becoming increasingly available are producing interchangeable information.

## Background

Measurement of changes in quality of life (QoL) has become a standard outcome variable in evaluating different therapeutic regimes in cancer [1-3]. Standardized, validated and reliable questionnaires are available for the measurement of changes in QoL [4-7]. Additionally, the use of these instruments by clinicians caring for patients is being explored [8-11]. Assessing changes in QoL as patients progress through the course of disease and treatment increases the need for longitudinal assessment.

Computer versions of these questionnaires have become available and can be used for longitudinal assessments [12-17]. These systems are well accepted by patients [12,16-19] and allow for the collection of data without transcription errors [12,17,18]. Comparison of data collected at one point in time by computer versus paper suggest that the method of collecting the information does not have a large effect on the data collected [19], although some differences are obtained. Formatting of the questions has been found to have an effect [20], and there may be a tendency of patients to give more positive responses with the computer, especially if the format is simplified [12,20].

A potential barrier to longitudinal measurements is that compliance may decrease over time. Patients may be initially willing to answer questions on the computer, but be less willing to do so on subsequent visits [14,17]. Reasons for this may include time constraints due to office and patients' scheduling needs as well as patients feeling unwell. One method to deal with these realities of daily clinical practice is to offer patients the choice of taking home the questionnaires to complete if they state they do not have time or do not want to complete the questionnaires on the computer at that time. This would introduce two principal differences. The method of data collection would be different (paper vs. computer), and the location of completing the forms would be different (home vs. office). Asking patients about their QoL over the past several days would reduce the effect of answering the questions in the office or at home. If the instruments are measuring significant changes in life due to major events such as diagnosis of serious disease, surgery, chemotherapy, and remission, then the location and method of administration of the instrument should have minimal effect on responses.

Women attending a gynecologic oncology practice were enrolled in a longitudinal study of QoL. Women completed a computer version of a QoL questionnaire pre-operatively and again at six months. They were given the option of using the paper version at either time point, and the effect of this choice was examined. An additional issue examined was whether use of the paper version was widespread or sporadic. The goal of the study was to compare changes over time obtained when women used a touch-screen computer on two occasions with changes obtained when women used a paper version of the questionnaire on one of the occasions.

## Methods

Patients who were scheduled to undergo surgery for endometrial cancer, ovarian cancer or an adnexal mass were invited to participate in a long term study of QoL, complementary medicine use and diet. Women at two gynecologic oncology offices in Northeast Ohio were recruited from 2001 – 2003. Informed consent was obtained for participation in this IRB approved study. Private office records and hospital discharge records were reviewed to abstract demographics and final pathology diagnosis. Baseline demographics were ascertained by interview with a research assistant pre-operatively. Patients completed the questionnaire pre-operatively and again at six months.

Computer kiosks with a 15 inch monitor were programmed with the Functional Assessment of Cancer Therapy-General questionnaire (FACT-G) along with an additional fatigue module [21]. The FACT is a 27-item questionnaire consisting of four domains: physical, emotional, social and functional well-being. Patients are asked how true each statement has been for them over the past seven days. Each domain is comprised of six to seven questions scored by use of a Likert-type scale ranging from 0 (not at all) to 4 (very much). Each domain appeared on one screen and patients touched their response to each individual question. Patients could change their answers by touching an alternate response on that screen but could not return to a previous screen. All questions had to be completed before the computer continued to the next screen. The touch screen computer was designed so that the format of the questions closely matched the format of the questions on the paper form. Patients utilized the computer kiosk independently during their office visit although the research assistant was available to answer questions. Patients were given the option of completing the questionnaire using the paper version at any time.

### Statistical analyses

Patients were categorized into four groups; those who completed the FACT-G on the computer on both occasions (CC), those who completed the initial assessment by computer and used paper format at six months (CP), patients who completed the initial assessment on paper and the six-month via computer (PC) and patients who completed both assessments on paper (PP). Analysis of variance or chi-square statistic was used to compare baseline demographic variables between patients who always used the computer and those who utilized the paper version at either time point.

Repeated measures analysis-of-variance was used to analyze change in the domain score from baseline to six months (time effect), whether there was an effect of group (CC, CP, PC and PP) and whether there was an interaction between group and time. Significance was set at p < 0.01 due to multiple comparisons. SPSS version 10.0 was used for analysis (Chicago, IL).

## Results

A total of 187 patients were asked to participate in this longitudinal study and 151 agreed (81%). Following completion of the initial assessment, 32 patients were lost to follow-up, moved, missed the second appointment entirely or refused to complete the questionnaire the second time (16 patients with benign adnexal mass, 8 with endometrial cancer and 8 with ovarian cancer). A total of 119 patients (79% of patients who agreed to participate in the study) completed the FACT-G assessments at both time points. Forty patients had endometrial cancer, 40 had ovarian cancer and 39 had a benign adnexal mass. Twenty of the cancer patients had Stage III or IV disease. Virtually all of the patients were Caucasian (96.6%).

Patients returned the questionnaire by mail within a few days of their scheduled visit. Seventy-one (60%) patients used the computer at both visits (CC), 26 (21.8%) used the computer initially followed by the paper version at six months (CP), 17 (14.3%) used the paper version initially followed by the computer version (PC), and five patients (4.2%) used the paper version at both visits (PP). Patients in the PP group were excluded from statistical analyses as the numbers in that group were small (n = 5).

There were no differences in the age (F = 0.225, p = 0.80) or level of education (χ^2 ^= 2.75, p = 0.60) between the CC, PC and CP groups (Table 1). Approximately 60% of the patients within each diagnosis group used the computer at both time points (Table 1). A slightly higher percentage of patients with a benign adnexal mass used the paper version of the FACT-G at the six months visit (χ^2 ^= 11.07, p = 0.026) as they were more likely to decline to come in for an office visit and request the FACT-G be sent home than were the patients with a cancer diagnosis (Table 1). Four of the five patients in the PP group had ovarian cancer. Mean age of those in the PP group was similar to the other groups (61.2 years) and all had some college or were college graduates.

**Table 1:** Patient Demographics by Group

	**CC (n = 71)**	**CP (n = 26)**	**PC (n = 17)**
**Age (mean ± SEM)**	58.3 ± 1.5	56.4 ± 2.6	57.4 ± 2.9
**Diagnosis**			
Benign (n = 39)	24 (61.5%)	14 (35.9%)	1 (2.6%)
Endometrial CA (n = 39)	25 (64.1%)	5 (12.8%)	9 (23.1%)
Ovarian CA (n = 36)	22 (61.1%)	7 (19.4%)	7 (19.4%)
			
**Education**			
HS or less	31 (43.7%)	14 (53.8%)	11 (64.7%)
Some college	14 (19.7%)	4 (15.4%)	2 (11.8%)
College grad or higher	26 (36.6%)	8 (30.8%)	4 (23.5%)

**Figure 1:**
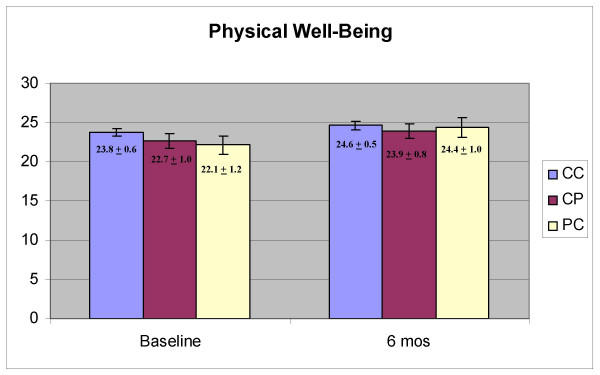
Scores on the Physical Well-Being domain of the Functional Assessment of Cancer Therapy (FACT-G)

**Figure 2:**
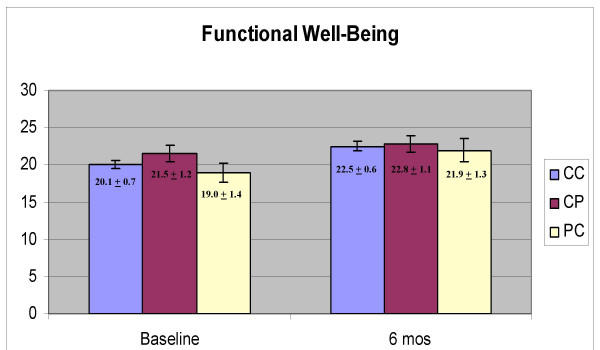
Scores on the Functional Well-Being domain of the Functional Assessment of Cancer Therapy (FACT-G)

**Figure 3:**
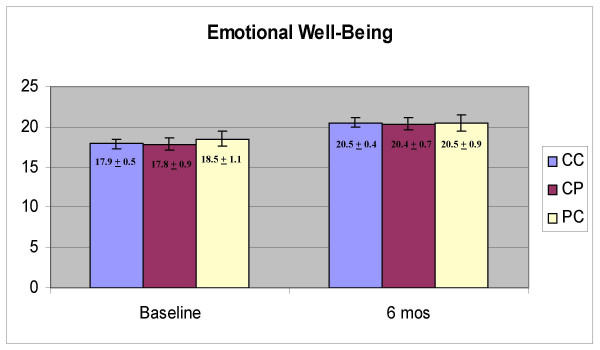
Scores on the Emotional Well-Being domain of the Functional Assessment of Cancer Therapy (FACT-G)

**Figure 4:**
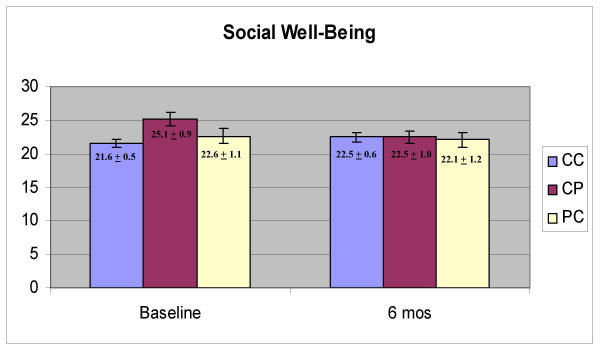
Scores on the Social Well-Being domain of the Functional Assessment of Cancer Therapy (FACT-G)

**Figure 5:**
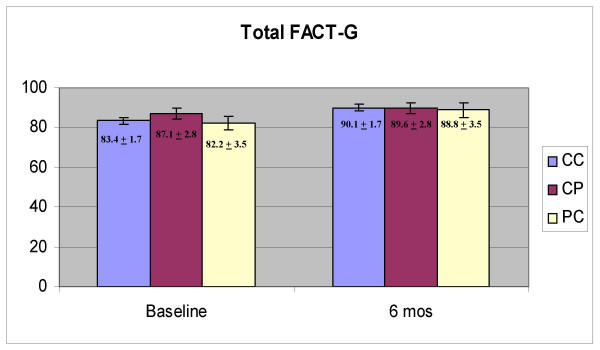
Total scores on the Functional Assessment of Cancer Therapy (FACT-G)

Physical well-being domain scores were significantly higher at six months than at baseline (Figure 1, F = 8.849, p = .004) and there was no effect of group (CC, CP, PC; p = 0.480) and no interaction between time and group (p = 0.457). Functional well-being scores were also higher at six months (Figure 2, F = 14.024, p < 0.001) and there was no effect of group (p = 0.453) and no interaction effect (p = 0.583). Emotional well-being scores were significantly higher at six months (Figure 3, F = 24.334, p < 0.001) and there was no effect of group (p = 0.943) and no interaction between group and time (p = 0.865). Social well-being scores did not increase with time (Figure 4, p = 0.14) and there was no effect of group (p = 0.185). There was a significant interaction between group and time (F = 5.671, p = 0.005) as the CP group had a higher score at baseline. There was no effect of time, group or interaction on fatigue scores (data not shown). Total scores were significantly higher at six months (Figure 5, F = 12.174, p = 0.001) and there was no effect of method (p = 0.756) and no interaction effect (p = 0.392).

## Discussion

Physical, functional, emotional well-being, and total scores, improved significantly between baseline and six months. In all cases, there was no effect of group and no interaction between group and time, indicating that the women were not affected by the method of data collection. There were also no significant effects of group even when there was no change in the scores over time (social well-being, fatigue). The one significant interaction effect was observed with the social well-being domain, which appeared due to a high baseline score in the CP group. At baseline, the CP group was the same as the CC group (they all used the computer) so it is not clear why there would be a high baseline score in the group that would use a paper version six months later. It is possible that with the number of tests conducted, one spurious finding would be obtained. The trend across all the tests is very strong, however. There are clear and significant changes with time but not with the method of obtaining the data.

Given the choice between using the computer version and the paper version, a small number of women chose the paper version. Of the 238 total measurements, the paper version was used a total of 53 times (22%). Reasons for not using the computer included not wanting to come in to the physician's office at all and patient preference but also instances beyond the patients' control such as scheduling complications and researcher unavailability on a small number of occasions. Designing strategies to increase computer availability may result in further reductions in patient use of the paper versions. If patients can log onto the computer using a unique identifier and complete the questionnaires on their own in the waiting room, the number of women who have to take questionnaires home or forgo completing them should decrease even further.

The second assessment occurred six months following major surgery for all women. The majority of women with ovarian cancer received chemotherapy, but were not receiving it at six months. This time point therefore allows a relatively stable point to assess changes in QoL relative to pre-operative scores in these groups of women. It is possible that differences in method of data collection would be obtained if women were acutely ill at the time of measurement, however the time frame of seven days used in the FACT-G reduces the likelihood that a separation in time of a day or two between using the computer in the office or the paper version at home will result in different responses. The time frame used in the FACT-G, and the relatively stable time point chosen may therefore contribute to the lack of measurement effect obtained in these groups of women.

A limitation of this study is the lack of minority representation which may reduce the generalizability of these results. Additionally, 19% of patients refused to participate in the study. Of the patients who did participate, 21% did not complete the second assessment, although this figure includes 16 women with a benign adnexal mass who may have returned to their referring physician, and women with cancer who moved or transferred their care. Nonetheless, the women who remained on study may differ from those who did not agree to participate or who did not complete the second assessment. They may, for example, have a greater degree of commitment to the research process.

A second limitation is that women were not randomly assigned to use either the computer or the paper versions. This is a preliminary examination of existing data to determine whether there appeared to be a selection bias, or major effect, of using the paper version. Women with QoL scores that differed markedly from the norm, for example, might have chosen to take the paper version home. This did not appear to be the case, however, as highly significant effects of time were observed, but group and interaction effects were markedly non-significant. Related limitations include the remote, but possible, explanation that the first method of administration had an effect on participants at the second time point. Additionally, patient choice itself may have had an unmeasured effect. For example, women with benign adnexal mass were more likely to forego the second office visit and complete the questionnaire at home. Disease and questionnaire administration are therefore confounded. These limitations may have influenced group choice, as well as responses on the second measurement.

These exploratory data suggest that women are responding to questionnaires presented on a computer in the same manner as questionnaires on paper. This study therefore differed somewhat from studies that found differences in method of administration [12,20]. An important consideration may be maintaining the same format of the questions in the two methods of administration. In this study, each domain was presented on one large screen so that all questions were listed together. The similarity of the format may have contributed to the finding that modes of administration are interchangeable, however larger scale studies, which include randomization and assessing women at different stages of treatment, should be conducted to verify these findings.

## Conclusions

Longitudinal measurements of health- related QoL are increasingly used in cancer patients. This study examined whether two different methods of measuring QoL (computer and paper) would provide interchangeable data. It appears that patients are dealing with issues of significant concern and they are responding to the content of the questions and not the method of data collection. It is clearly desirable to standardize the method of data collection and have conditions remain constant across time. The results of this study, however, demonstrate that valid data are obtained with alternate methods of data collection and this is preferable to foregoing data collection entirely.

## Authors' contributions

KG and VVG conceived of the study, and participated in its design and coordination. HF, MH, EJ and VVG implemented the study and were responsible for day to day conduct of the study. KG and HF analyzed the data. KG, HF, VVG drafted the manuscript; JE and MH provided critical review. All authors read and approved the final manuscript.

## References

[B1] Schwartz CE, Sprangers MA (2002). An introduction to quality of life assessment in oncology: the value of measuring patient-reported outcomes. Am J Manag Care.

[B2] Boling W, Fouladi RT, Basen-Engquist K (2003). Health-related quality of life in gynecological oncology: instruments and psychometric properties. Int J Gynecol Cancer.

[B3] Osoba D (1999). What has been learned from measuring health-related quality of life in clinical oncology. Eur J Cancer.

[B4] Cella DF, Tulsky DS, Gray G, Sarafian B, Linn E, Bonomi A, Silberman M, Yellen SB, Winicour P, Brannon J (1993). The Functional Assessment of Cancer Therapy scale: development and validation of the general measure. J Clin Oncol.

[B5] Basen-Engquist K, Bodurka-Bevers D, Fitzgerald MA, Webster K, Cella D, Hu S, Gershenson DM (2001). Reliability and validity of the functional assessment of cancer therapy-ovarian. J Clin Oncol.

[B6] Aaronson NK, Ahmedzai S, Bergman B, Bullinger M, Cull A, Duez NJ, Filiberti A, Flechtner H, Fleishman SB, de Haes JC (1993). The European Organization for Research and Treatment of Cancer QLQ-C30: a quality-of-life instrument for use in international clinical trials in oncology. J Natl Cancer Inst.

[B7] Kemmler G, Holzner B, Kopp M, Dunser M, Margreiter R, Greil R, Sperner-Unterweger B (1999). Comparison of two quality-of-life instruments for cancer patients: the functional assessment of cancer therapy-general and the European Organization for Research and Treatment of Cancer Quality of Life Questionnaire-C30. J Clin Oncol.

[B8] Detmar SB, Muller MJ, Schornagel JH, Wever LD, Aaronson NK (2002). Health-related quality-of-life assessments and patient-physician communication: a randomized controlled trial. JAMA.

[B9] Taenzer P, Bultz BD, Carlson LE, Speca M, DeGagne T, Olson K, Doll R, Rosberger Z (2000). Impact of computerized quality of life screening on physician behaviour and patient satisfaction in lung cancer outpatients. Psychooncology.

[B10] Velikova G, Brown JM, Smith AB, Selby PJ (2002). Computer-based quality of life questionnaires may contribute to doctor-patient interactions in oncology. Br J Cancer.

[B11] Velikova G, Booth L, Smith AB, Brown PM, Lynch P, Brown JM, Selby PJ (2004). Measuring quality of life in routine oncology practice improves communication and patient well-being: a randomized controlled trial. J Clin Oncol.

[B12] Velikova G, Wright EP, Smith AB, Cull A, Gould A, Forman D, Perren T, Stead M, Brown J, Selby PJ (1999). Automated collection of quality-of-life data: a comparison of paper and computer touch-screen questionnaires. J Clin Oncol.

[B13] McLachlan SA, Allenby A, Matthews J, Wirth A, Kissane D, Bishop M, Beresford J, Zalcberg J (2001). Randomized trial of coordinated psychosocial interventions based on patient self-assessments versus standard care to improve the psychosocial functioning of patients with cancer. J Clin Oncol.

[B14] Wright EP, Selby PJ, Crawford M, Gillibrand A, Johnston C, Perren TJ, Rush R, Smith A, Velikova G, Watson K, Gould A, Cull A (2003). Feasibility and compliance of automated measurement of quality of life in oncology practice. J Clin Oncol.

[B15] Carlson LE, Speca M, Hagen N, Taenzer P (2001). Computerized quality-of-life screening in a cancer pain clinic. J Palliat Care.

[B16] Buxton J, White M, Osoba D (1998). Patients' experiences using a computerized program with a touch-sensitive video monitor for the assessment of health-related quality of life. Qual Life Res.

[B17] Allenby A, Matthews J, Beresford J, McLachlan SA (2002). The application of computer touch-screen technology in screening for psychosocial distress in an ambulatory oncology setting. Eur J Cancer Care (Engl).

[B18] Drummond HE, Ghosh S, Ferguson A, Brackenridge D, Tiplady B (1995). Electronic quality of life questionnaires: a comparison of pen-based electronic questionnaires with conventional paper in a gastrointestinal study. Qual Life Res.

[B19] Taenzer PA, Speca M, Atkinson MJ, Bultz BD, Page S, Harasym P, Davis JL (1997). Computerized quality-of-life screening in an oncology clinic. Cancer Pract.

[B20] Boyes A, Newell S, Girgis A (2002). Rapid assessment of psychosocial well-being: are computers the way forward in a clinical setting?. Qual Life Res.

[B21] Yellen SB, Cella DF, Webster K, Blendowski C, Kaplan E (1997). Measuring fatigue and other anemia-related symptoms with the Functional Assessment of Cancer Therapy (FACT) measurement system. J Pain Symptom Manage.

